# Comparison of luteal phase stimulation with follicular phase stimulation in poor ovarian response: a single-blinded randomized controlled trial

**DOI:** 10.1186/s40834-024-00265-z

**Published:** 2024-02-18

**Authors:** Mozhgan Vahabi Dastjerdi, Soheila Ansaripour, Mina Ataei, Roya Gharedaghi, Seyedeh Melika Mostafavi Hoseini, Arash Mohazzab, Simin Zafardoust

**Affiliations:** 1 Reproductive Biotechnology Research Center, Avicenna Research Institute, ACECR, Tehran, Iran; 2 Department of Obstetrics and Gynecology, Social Determinants of Health, Research Center, School of Medical Sciences, Alborz University of Medical Sciences, Karaj, Iran; 3 Department of Epidemiology, School of Public Health, Iran University of Medical Sciences, Tehran, Iran; 4 Reproductive Immunology Research Center, Avicenna Research Institute, ACECR, Tehran, Iran

**Keywords:** Ovarian stimulation, Poor response, Follicular phase, Luteal phase

## Abstract

**Background:**

In the last decade, luteal-phase ovarian stimulation (LPOS) has been suggested as an alternative controlled ovarian stimulation (COS) protocol for in vitro fertilization/intracytoplasmic sperm injection (IVF/ICSI) cycles mainly in women with a history of poor ovarian response (POR). The present randomized controlled trial study aimed to compare the outcomes of follicular phase ovarian stimulation (FPOS) and LPOS protocols in POR cases undergoing ICSI cycles.

**Methods:**

Seventy-eight POR patients who met the Bologna criteria and underwent an ICSI cycle were included. In this study, 39 POR cases were allocated to the FPOS group, and 39 POR cases were allocated to the LPOS group. The primary outcome was the number of metaphase II (MII) oocytes. In addition, the total number of oocytes, number of top-quality day 3 embryo, day 3 embryo development rate, chemical pregnancy and clinical pregnancy rates were defined as secondary outcomes.

**Results:**

The obtained results demonstrated that the number of MII oocytes significantly increased in the LPOS group compared to the FPOS group (*P* = 0.007). However, there was no significant difference between the two groups regarding the number of GV and MI oocytes, number of top-quality day 3 embryos and day 3 embryo development rate among both categories of patients. Also, the number of total and MII oocytes was significantly higher in the LPOS group (*P* = 0.016).

**Conclusion:**

These results suggest that LPOS protocol effectively increases the number of mature oocytes in women with a history of POR.

**Trial registration:**

IRCT20210405050852N1 (Registered at Iranian registry of clinical trials; available at https://en.irct.ir/trial/55402).

## Background

The ovarian response is a critical factor of effectively controlled ovarian stimulation (COS) during the treatment of infertile patients undergoing in vitro fertilization/intracytoplasmic sperm injection (IVF/ICSI) cycles [1–4]. Poor ovarian response (POR) in 9–24% of IVF/ICSI cycles, leads to insufficient retrieval of mature oocytes, cycle cancellation and low pregnancy rates [1, 5]. According to the Bologna criteria published by the European Society of Human Reproduction and Embryology (ESHRE) in 2011, POR is identified with at least two of the three following criteria: (1) advanced maternal age (≥ 40 years) or any other risk factor for POR, (2) a previously characterized POR cycle (≤ 3 oocytes with a conventional stimulation protocol), (3) an abnormal ovarian reserve test (antral follicle count (AFC) < 5–7 follicles or anti mullerian hormone (AMH) < 0.5–1.1 ng/mL) [6].

Various protocols have been established to improve ovarian response in POR cases. Nevertheless, the practical and applicable strategy for these women remains controversial [7]. Standard ovarian stimulation protocols in IVF/ICSI cycles are typically initial from the early follicular phase of the menstrual cycle. However, follicular phase ovarian stimulation (FPOS) may cause several complications, such as ovarian hyperstimulation syndrome, suboptimal oocyte quality and premature luteinization [8, 9]. Hence, the question arises whether IVF/ICSI with conventional FPOS has alternative methods, especially in the POR cases. Current evidence specifies that folliculogenesis occurs in a wave-like mode. This finding indicates that there are various follicular recruitment waves in the same menstrual cycle [10, 11]. Therefore, the conventional concept that a sole cohort of antral follicles only grows during the follicular phase of the menstrual cycle is debated [12]. In recent years, luteal phase ovarian stimulation (LPOS) has been recognized as an acceptable method for attaining an adequate number of competent oocytes in the shortest period [13].

LPOS was primarily used for fertility preservation in cancer patients and then applied in the general population of infertile couples [14]. Studies have disclosed similar numbers of mature oocytes and comparable fertilization rates in LPOS and FPOS protocols in cases with normal ovarian response [14, 15]. It was shown that the LPOS protocol improved IVF/ICSI outcomes compared to the FPOS protocol in women with a history of POR [9, 16]. The conceivable reasoning is that physiologically high levels of progesterone in the luteal phase could effectively block a premature luteinizing hormone (LH) surge that more regularly happens in POR patients during ovarian stimulation [9, 16]. Based on current evidence, it seems that an innovative protocol of LPOS could be considered a better regimen for managing POR cases which leads to the harvest of more capable oocytes and embryos compared to FPOS [10, 11]. However, further randomized controlled studies are necessary to approve the effectiveness of LPOS in POR cases and to inspect a perfect LPOS protocol. In this regard, the present randomized controlled trial study aimed to compare the clinical outcomes of FPOS and LPOS protocols in POR cases undergoing ICSI cycles.

## Methods

### Design and settings

The present single-blinded randomized controlled trial study was carried out on cases with POR referred to Avicenna Infertility Clinic, Tehran, Iran, between 2021-07-01 and 2021-12-31. Written informed consent was obtained from all the participants. The trial was registered at the Iranian Registry of Clinical Trials (IRCT20210405050852N1). POR patients who met the Bologna criteria and underwent FET cycles were entered into the study based on the following inclusion/exclusion criteria: Women with infectious diseases, sexually transmitted diseases, autoimmune disorders, tubal factor infertility, endometriosis, chronic inflammatory diseases, hormonal or anatomical disorders, endometriosis, presence of space-occupying lesions, history of ectopic pregnancy or miscarriage, myomas, polyps, adhesions, previous pelvic surgeries, cancer diagnosis, thrombophilic disorders, anemia and body mass index (BMI) ≥ 30 kg/m2 were all excluded. In addition, participants with chromosomal abnormalities and severe male factors of their spouses were excluded. History of one ICSI failed cycle with less than four oocytes and AMH < 1.1 ng/ml were deemed inclusion criteria.

### Randomization

Randomization was performed using simple block randomization with sealedenvelop.com software, through the block size of four. The random sequence was concealed from the principal investigator. It was only available for an independent third person and was revealed individually during the study period. POR patients who met the Bologna criteria and underwent an ICSI-frozen embryo transfer (FET) cycle were assessed for their suitability to enter one of two groups including the FPOS group (*N* = 39) or the LPOS group (*N* = 39). This was a single-blinded study. The participants were not aware of the type of treatment in each group. To blind the patients participating in this study, all conditions were the same between the two groups, so patients in the intervention and control groups were referred to the center on ovulation stimulation days in both groups.

### Procedures

#### Follicular phase ovarian stimulation

Follicular phase ovarian stimulation was conducted using GnRH antagonist protocol. Briefly, women underwent gonadotropin stimulation using follitropin α (Cinnal-f®, CinnaGen, Iran) at a dose of 300 IU/day and human menopausal gonadotropin (HMG) (Menotropin®, CinnaGen, Iran) at a dose of 150–225 IU/day beginning from day 2–3 of the menstrual cycle. When the diameter of the follicles reached 12 mm, GnRH antagonist (0.25 mg/day; Cetrotide®, Merck Serono, Germany) was injected and sustained until ovulation induction. As soon as the diameter of one or more follicles was > 18 mm on transvaginal ultrasound, 250 µg of recombinant human chorionic gonadotropins (hCG) (Ovitrelle®, Merck Serono, Germany) was administered to initiate the ovulation induction.

#### Luteal phase ovarian stimulation

In the LPOS group, transvaginal sonography established natural ovulation between day 15 and day 18 of the menstrual cycle. Natural ovulation was confirmed when transvaginal sonography revealed a lack of dominant follicles. After approval of spontaneous ovulation, the women with a minimum of one follicle of less than 8 mm underwent ovarian stimulation with 300 IU/day of follitropin α (Cinnal-f®, CinnaGen, Iran), 150–225 IU/day of HMG (Menotropin®, CinnaGen, Iran) as well as 10 mg/day medroxyprogesterone (Aburaihan Co., Tehran, Iran). When the leading follicle extended 14 mm, the women received GnRH antagonist (0.25 mg/day; Cetrotide®, Merck Serono, Germany) until the day of oocyte trigger. Ovulation induction was conducted using 250 µg of hCG (Ovitrelle®, Merck Serono, Germany) when the leading follicle developed larger than 18 mm.

#### Assisted reproductive techniques

Ovum pick-up (OPU) was conducted transvaginally 36 h after hCG was injected. Cumulus cell-oocyte complexes (COCs) were retrieved and washed in MOPS-buffered medium (G-MOPS™ PLUS, Vitrolife Co., Sweden). Oocyte denudation was performed 2 h after retrieval utilizing hyaluronidase (HYASE-10X™, Vitrolife Co., Sweden) followed by mechanical dissection. ICSI was conducted on all mature metaphase II oocytes 3–4 h after OPU. Then metaphase II oocytes cultured in an embryo culture medium (SAGE 1-Step™, CooperSurgical Co., USA) until day 3. The embryo culture was conducted in an incubator with a humidified atmosphere and 6% CO2.

Day 3 embryo quality was evaluated in the previous literature [17]. Top-quality day 3 embryos were determined as those with 8–10 symmetric blastomeres on day 3, < 15% fragmentation, absence of multinucleation, and absence of intracytoplasmic and extra-cytoplasmic abnormalities. Otherwise, the embryos were considered as low-quality embryos. In addition, the day 3 embryo development rate was measured as the number of 8-cell embryos on day 3 per number of normally fertilized oocytes × 100 [18].

#### FET cycle

Day 3 embryos were warmed in the commercial media (Kitazato BioPharma Co., Shizuoka, Japan) based on the manufacturer’s protocol. After the warming procedure, the embryos were located in an embryo culture medium (SAGE 1-Step™, CooperSurgical Co., USA) and incubated at 37 °C in 6% CO2 until the embryo transfer (ET) procedure. Embryo transfer (ET) was conducted using an embryo transfer catheter (Cook, USA) by an expert gynecologist under the guidance of ultrasound, based on the guidelines provided by the American Society for Reproductive Medicine (ASRM). Top-quality day 3 embryo was selected for each FET cycle. Endometrial preparation was conducted using 6 mg/d orally estradiol valerate (Aburaihan Co., Tehran, Iran) from the second (or third) day of the menstrual cycle for 14 days plus progesterone (400 mg, suppository, BID; Cyclogest, Actavis, England, UK) 5 days before ET until the 12th week of pregnancy.

### Outcomes

The primary outcome was the number of metaphase II (MII) oocytes. In addition, the total number of oocytes, number of top-quality day 3 embryo, day 3 embryo development rate, chemical pregnancy and clinical pregnancy rates were defined as secondary outcomes.

The chemical pregnancy rate was determined by the number of pregnancies diagnosed by positive serum β-hCG (b-hCG > 50mIU/ml) after two weeks from the day of ET per number of FET cycles × 100.

The clinical pregnancy rate was calculated by the number of pregnancies with a heartbeat of one or more confirmed by ultrasound after six weeks from the day of ET per number of FET cycles × 100.

### Statistical analysis

The results were shown as median and range. Outcomes were compared between two groups of study using independent t-test, U Mann Whitney (for non-parametric variables) and Chi-squared and Fisher exact test (for categorical variables). To adjust the effect of POR severity on the embryologic outcomes, a comparison of the oocyte retrieval between two groups was performed by analysis of covariance (ANCOVA) considering serum AMH level and number of oocytes in previously performed COH cycle. SPSS software version 22 (IBM Software, USA) was used for analysis. The results were analyzed with an intention-to-treat and per protocol (on patients who underwent Oocyte pick up) approach. Diagrams were created using GraphPad Prism (GraphPad Software, USA). The *p* < 0.05 was considered as statistically significant.

## Results

**Fig. 1:**
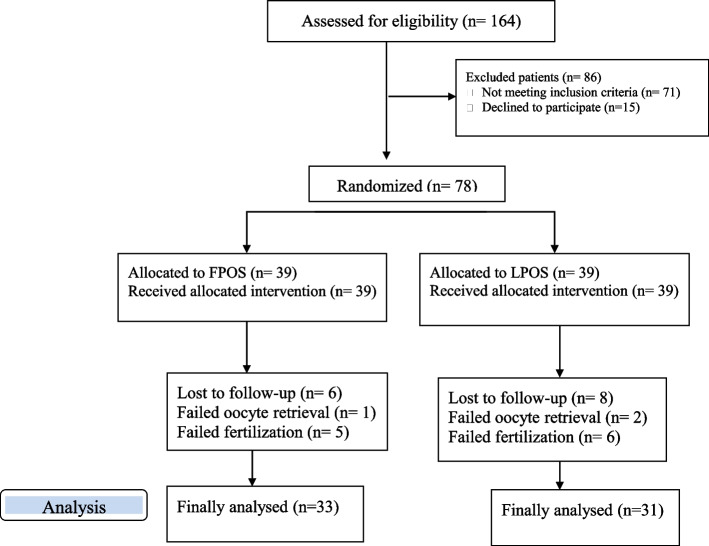
Consort flow diagram

One hundred sixty-four participants were assessed for eligibility to enter the study, from which 78 patients fulfilled the inclusion criteria and were enrolled. Finally, 14 patients (6 patients in the FPOS group and 8 patients in the LPOS group) were left out for different causes, 64 couples accomplished the trial and their data were analyzed (Fig. 1). There was no significant difference between the two groups in baseline characteristics, including age, body mass index (BMI), serum level of day 3 follicle-stimulating hormone (FSH), antral follicle count (AFC), gravidity and parity. In addition, there was no significant difference between the two groups in the total number of gonadotropin ampoules (75 IU) and duration of gonadotropin administration in the previous cycle. However, the serum level of anti-Mullerian hormone (AMH) was significantly higher in the FPOS group compared to the LPOS group. Table 1 represents the demographic and clinical characteristics of the studied groups.

**Table 1:** Demographic and clinical characteristics of the studied groups

Variable	LPOS, *N* = 31	FPOS, *N* = 33	*P* value
Age (year)	37 (26–39)	38 (30–39)	0.86
BMI	26 (21.4–34)	25 (20-26.2)	0.2
FSH (Day 3) (mIU/ml)	9 (3–16)	9 (3–19)	0.80
AMH (ng/ml)	0.5 (0.1–1.3)	0.9 (0.2–1.5)	0.037*
AFC	3 (1–5)	3 (2–5)	0.762
Gravidity	0 (0)	0 (0–2)	0.92
Parity	0 (0–1)	0 (0–1)	0.088
Number of gonadotropin ampoule (75 IU) (previous cycle)	67.3 (35–120)	61.7 (30–104)	0.23
Days of gonadotropin administration (previous cycle)	9.18 (5–12)	9.32 (6–14)	0.76
Number of previous IVF failure	1 (1–6)	1 (1–3)	0.362

*AFC *Antral Follicle Count, *AMH* Anti-Mullerian hormone, *BMI* Body mass index, *FPOS *follicular phase ovarian stimulation, *FSH* Follicle Stimulating Hormone, *MI* Metaphase-I, *MII* Metaphase-II, *LPOS* Luteal-phase ovarian stimulation. The results were shown as median and range

* Significant

*P-value < 0.05

### Primary and secondary outcomes of the studied groups

**Fig. 2:**
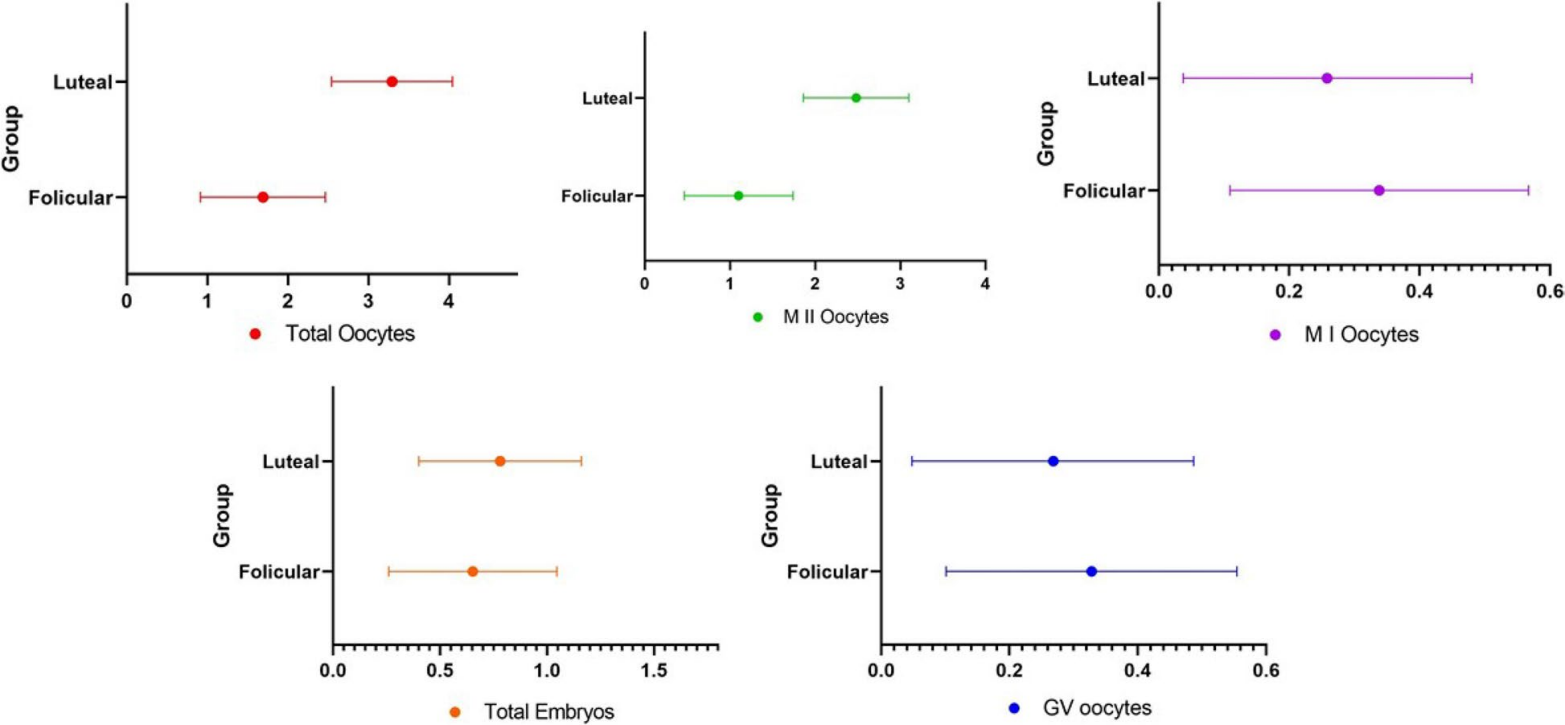
Outcomes of assisted reproductive technology (ART) between two study groups after adjustment with anti-Mullerian hormone (AMH) and results of last previous cycle

Table 2 represents the result of primary and secondary outcomes of the studied groups, including the total number of oocytes, number of germinal vesicle (GV), metaphase I (MI) and MII oocytes, number of top-quality day 3 embryos and day 3 embryo development rate. The obtained data were analyzed based on patients who intended to receive treatment and those who underwent oocyte pick-up. Based on the obtained results, there was no significant difference between the two groups in terms of the number of GV and MI oocytes, the number of top-quality day 3 embryos and the day 3 embryo development rate among both categories of patients. However, number of MII oocytes (*p*-value = 0.007) was significantly higher in the LPOS group compared to the FPOS group in patients who intended for treatment. Further analysis using adjustment of ART outcomes with AMH and results of the last previous cycle showed that the number of total and MII oocytes were significantly higher in the LPOS group (*P* = 0.007, 0.016 respectively) (Fig. 2).

**Table 2:** Primary and secondary outcomes of assisted reproductive technology (ART) in the studied groups by two different analytical approaches

Variable	Intention to treat			Undergoing oocyte pick-up		
	LPOS, *N* = 31	FPOS, *N* = 33	*P* value	LPOS, *N* = 23	FPOS, *N* = 25	*P* value
Total number of oocytes retrieved	3 (0–8)	2.5 (0–6)	0.078	4 (0–8)	4 (0–6)	0.081
Number of MII oocyte	3 (0–8)	2 (0–5)	0.041*	3 (0–8)	2 (0–5)	0.07
Number of MI oocyte	0 (0–2)	0 (0–2)	0.539	0 (0–2)	1 (0–2)	0.518
Number of GV oocyte	0 (0–2)	0 (0–3)	0.623	0 (0–2)	0 (0–3)	0.559
Day 3 embryo development rate	45.5%	38.7%	0.585	60%	52.2%	0.196
Top-quality day 3 embryo	42.4%	25.8%	0.162	56.8%	34.8%	0.141

*FPOS* follicular phase ovarian stimulation, *MI* Metaphase-I, *MII* Metaphase-II, *LPOS* Luteal-phase ovarian stimulation. The results were shown as median and range

* Significant

*P-value < 0.05

The overall comparison of clinical outcomes between the two study groups is presented in Table 3. Embryo transfer (ET) was conducted for 11 patients in the FPOS group and 11 patients in the LPOS group. There was no significant difference in terms of chemical pregnancy and clinical pregnancy rate in the studied groups. The chemical pregnancy rate was 27.3% (3/11) for the FPOS group and 9.1% (1/11) for the LPOS group. The clinical pregnancy rate was 9.1% (1/11) for the LPOS group. Clinical pregnancy was not identified in the FPOS group.

**Table 3:** Clinical outcomes analysis of the studied groups

Variable	LPOS, *N* = 11	FPOS, *N* = 11	*P* value
Chemical Pregnancy rate	3 (27.3%)	1 (9.1%)	0.306
Clinical Pregnancy rate	1 (9.1%)	0 (0%)	0.500

*FPOS* follicular phase ovarian stimulation, *LPOS* Luteal-phase ovarian stimulation

## Discussion

Increasing oocyte yield, viable embryo production, and pregnancy probability are the main goals of stimulation protocols. It is well known that POR patients experience lower rates of pregnancy and more cycle cancellations. To increase these patients’ chances of becoming pregnant, several strategies have been put forth, but none have shown promise [12]. Because of their experience encouraging patients to preserve their fertility urgently (e.g. owing to the cancer diagnosis), it has been demonstrated that luteal phase stimulation is a practical method for obtaining mature oocytes and embryos for cryopreservation. Furthermore, because DuoStim stimulates the ovaries twice, once during the follicular phase and again during the luteal phase, a few days after oocyte retrieval, it offers the possibility to obtain more oocytes in a single menstrual cycle [19].

Today, patients undergoing IVF can maximize the number of oocytes retrieved in the shortest amount of time by using a newly developed protocol called DuoStim, which combines follicular and luteal phase stimulations during the same ovarian cycle. In POR patients, DuoStim was assessed in the majority of published studies [20, 21]. DuoStim’s approach is very promising because of its strengths. Both stimulations carried out in the luteal and follicular phases resulted in competent oocytes, with comparable rates of euploidy, blastulation, and fertilization. Additionally, the clinical outcomes following the transfer of a single euploid blastocyst were also similar. The following are some advantages of DuoStim: more patients may obtain a (chromosomally normal) blastocyst per ovarian cycle; no discernible difference in competence has been observed to date between oocytes obtained after FPOS and LPOS; the likelihood of obtaining at least one viable embryo in a single menstrual cycle may be increased; and the time required to obtain oocytes after FPOS and LPOS may be shortened. Sequential FPOS cycles with a low drop-out rate may not be as well-tolerated by patients as the DuoStim protocol [22].

We know that LPOS is a workable protocol for infertile patients. An early luteinizing hormone surge may be physiologically inhibited by high progesterone levels during the luteal phase, which could benefit PORs but poor IVF outcomes could arise from premature luteinization during FPOS in the conventional IVF protocol [23–26]. Our objective was to evaluate the clinical results of the LPOS and FPOS protocols in PORs undergoing in vitro fertilization. In a population of women with POR who meet Bologna criteria, this randomized controlled study compares the effectiveness of FPOS and LPOS. For patients who have been diagnosed with POR or who are elderly, this can therefore shorten the time required to obtain the greatest number of oocytes or embryos in the shortest amount of time. Our study also aims to evaluate LPOS independently of double ovarian stimulation in order to prevent any potential priming effect from the previous stimulation during the follicular phase of the same cycle.

The present study revealed that several mature oocytes significantly increased after luteal phase ovarian stimulation compared to follicular phase ovarian stimulation in women with a history of POR. However, there was no significant difference between the two groups regarding the number of GV and MI oocytes, number of top-quality day 3 embryos, day 3 embryo development rate, and chemical and clinical pregnancy rates.

The wave concept of follicle growth suggests that antral follicles at the luteal phase may not retreat, but instead develop into mature oocytes after stimulation. This phenomenon has been confirmed in animals and humans [16, 27]. In the luteal phase, the secretion of progesterone and inhibin A from the corpus luteum can prevent the development of a dominant follicle; however, exogenous gonadotropin can persuade the concurrent growth of a group of follicles [16]. Therefore, ovarian stimulation in the luteal phase induces synchronous follicular cohort recruitment in contrast to the conventional protocols, which typically initial throughout the menstrual period and lead to the nonsynchronous development of follicles. Consequently, LPOS possibly achieves more mature oocytes within a short period [12].

So outcomes of DuoStim in POR patients recommend an improved response in the second stimulation during the luteal phase; nevertheless, this consequence could be interpreted by priming stimulation in the follicular phase [20]. The progesterone and estradiol reach a high level, and FSH receptors increase in granulosa cells after FPOS resulting in a better response to ovarian stimulation and synchronizing the antral follicles that will develop during LPOS [21].

The efficacy of LPOS compared with FPOS in POR cases in separate cycles is not understood correctly. A published case-control study in 40 patients indicates that women with POR experiencing LPOS had comparable numbers of oocytes retrieved with those experiencing FPOS [20, 28]. A randomized study showed that LPOS has similar efficacy to FPOS and proposed that it might increase ovarian response in young individuals with POR [13]. Recently Chen et al., showed that the number of retrieved metaphase II oocytes, fertilized oocytes, day-3 embryos and top-quality day-3 embryos, clinical pregnancy rates and live birth rates were similar between LPOS and FPOS groups [11]. In contrast with the mentioned studies, Wei et al. demonstrated that the LPOS protocol has similar efficacy to FPOS and could be a superior method for POR which can increase the numbers of retrieved oocytes and transferable embryos [9]. In line with this study, Jochum et al. in a retrospective cohort study among women experiencing ovarian stimulation for fertility preservation specified that LPOS versus FPOS leads to obtaining a significant amount of total oocytes. In addition, they showed no difference in total doses of gonadotropin between the two groups [29]. The efficacy of LPOS in the IVF/ICSI procedures of women with a history of POR remains controversial despite several studies in this regard. Some studies recommended that LPOS could increase ovarian response to gonadotropins, improve the number of MII oocytes and consequently increase clinical outcomes [9, 29] as Lin et al. in 2018 demonstrated in 2018 that the LPOS group had a significantly higher number of retrieved oocytes, metaphase II oocytes, fertilized oocytes, and day-3 embryos than the FPOS group [30]. However, they were unable to detect any discernible variations in the rates of clinical pregnancies, ongoing pregnancies, abortions, and cancellations.

Some studies did not support LPOS as an alternative COS protocol in IVF/ICSI procedures because the clinical outcomes were not increased, even though some benefits might have been achieved through the use of this protocol [11, 28]. In this regard, the present study demonstrated that LPOS improved the total number of mature oocytes; however, it did not improve other ART outcomes compared to the FPOS approach.

POR represents a heterogeneous population. The young subpopulation has a better clinical prognosis regarding the clinical pregnancy rate [31]. Epidemiological studies showed that the clinical pregnancy rate in POR women undergoing ART was almost 18.% [32]. Most studies compared clinical pregnancy of the LPOS to DuoStim, and there is rare data regarding the effect of the LPOS approach on clinical pregnancy rate in separate cycles in POR cases. Wei et al., revealed that the LPOS protocol increased the clinical pregnancy rate compared to the FPOS protocol [9]. In the present study, the clinical pregnancy rate was 9.1% in the LPOS group and no clinical pregnancy was identified in the FPOS group. Although the rates of chemical and clinical pregnancy were higher in the LPOS group, these differences were not significant between the two groups. It is important to note that 11 embryo transfers were conducted in both groups. Therefore, the patient population was low and likely inadequate to interpret the possibly relevant effects of the LPOS approach on pregnancy outcomes.

In the present study, a sample size of 64 participants was estimated to be appropriate to evaluate the effectiveness of LPOS compared to FPOS based on former studies [13, 33]. Since women who meet the Bologna criteria contain a rare group of patients undergoing ICSI including large POR cases in the study was exceptionally tough. Despite the strict randomization procedure, women allocated to the FPOS group had greater AMH levels, and a better response was anticipated in this group, which in fact, did not happen.

The main strength of the present study is using similar medicine brands, doses, and stimulation protocols for FPOS and LPOS to avoid possible preconceptions due to the use of different regimes. However, designing the study as a single-blinded, single-center study with a small sample size is the limitation of the present report, which should be considered in interpreting the results. Therefore, more studies must be conducted in the future to approve the efficiency and safety of LPOS, in terms of pregnancy complications and peri-natal and post-natal outcomes.

## Conclusion

In conclusion, the present study provides evidence that more MII oocytes can be retrieved after LPOS than after the FPOS approach in POR patients. The LPOS-derived embryos showed similar competence and clinical outcomes as FPOS-derived ones.

## Data Availability

No datasets were generated or analysed during the current study.
